# Imidazothiazole Derivatives Exhibited Potent Effects against Brain-Eating Amoebae

**DOI:** 10.3390/antibiotics11111515

**Published:** 2022-10-30

**Authors:** Ruqaiyyah Siddiqui, Mohammed I. El-Gamal, Anania Boghossian, Balsam Qubais Saeed, Chang-Hyun Oh, Mohammed S. Abdel-Maksoud, Ahmad M. Alharbi, Hasan Alfahemi, Naveed Ahmed Khan

**Affiliations:** 1 College of Arts and Sciences, American University of Sharjah, Sharjah 26666, United Arab Emirates; 2 Department of Medical Biology, Faculty of Medicine, Istinye University, Istanbul 34010, Turkey; 3 Department of Medicinal Chemistry, College of Pharmacy, University of Sharjah, Sharjah 27272, United Arab Emirates; 4 Sharjah Institute for Medical Research, University of Sharjah, Sharjah 27272, United Arab Emirates; 5 Department of Medicinal Chemistry, Faculty of Pharmacy, Mansoura University, Mansoura 35516, Egypt; 6 Department of Clinical Sciences, College of Medicine, University of Sharjah, Sharjah 27272, United Arab Emirates; 7 Center of Biomaterials, Korea Institute of Science & Technology (KIST School), Seongbuk-gu, Seoul 02792, Korea; 8 Department of Biomolecular Sciences, University of Science & Technology (UST), Yuseong-gu, Daejeon 34113, Korea; 9 Medicinal & Pharmaceutical Chemistry Department, Pharmaceutical and Drug Industries Research Division, National Research Centre (NRC), Dokki, Giza 12622, Egypt; 10 Department of Clinical Laboratory Sciences, College of Applied Medical Sciences, Taif University, Taif 21944, Saudi Arabia; 11 Department of Medical Microbiology, Faculty of Medicine, Al-Baha University, Al-Baha 65799, Saudi Arabia

**Keywords:** *Naegleria fowleri*, amoebicidal, anti-amoebic, mortality, cytotoxicity, cytopathogenicity

## Abstract

*Naegleria fowleri (N. fowleri)* is a free-living, unicellular, opportunistic protist responsible for the fatal central nervous system infection, primary amoebic meningoencephalitis (PAM). Given the increase in temperatures due to global warming and climate change, it is estimated that the cases of PAM are on the rise. However, there is a current lack of awareness and effective drugs, meaning there is an urgent need to develop new therapeutic drugs. In this study, the target compounds were synthesized and tested for their anti-amoebic properties against *N. fowleri.* Most compounds exhibited significant amoebicidal effects against *N. fowleri*; for example, **1h**, **1j**, and **1q** reduced *N. fowleri’s* viability to 15.14%, 17.45% and 28.78%, respectively. Furthermore, the majority of the compounds showed reductions in amoeba-mediated host death. Of interest are the compounds **1f**, **1k**, and **1v**, as they were capable of reducing the amoeba-mediated host cell death to 52.3%, 51%, and 56.9% from 100%, respectively. Additionally, these compounds exhibit amoebicidal properties as well; they were found to decrease *N. fowleri’s* viability to 26.41%, 27.39%, and 24.13% from 100%, respectively. Moreover, the MIC_50_ values for **1e**, **1f**, and **1h** were determined to be 48.45 µM, 60.87 µM, and 50.96 µM, respectively. Additionally, the majority of compounds were found to exhibit limited cytotoxicity, except for **1l**, **1o**, **1p**, **1m**, **1c**, **1b**, **1zb**, **1z**, **1y**, and **1x**, which exhibited negligible toxicity. It is anticipated that these compounds may be developed further as effective treatments against these devastating infections due to brain-eating amoebae.

## 1. Introduction

*Naegleria fowleri (N. fowleri)* is a free-living protist of the Heterolobosea class; it is responsible for a fatal central nervous infection (CNS) known as primary amoebic meningoencephalitis (PAM); hence, it has been given the name brain-eating amoeba [1,2,3,4]. This amoeba is distributed across every continent except Antarctica [1]. The presence of *N. fowleri* in water makes individuals pursuing water related activities more susceptible; activities such as swimming, diving, water skiing, nasal cleansing, and the performance of ritual ablutions all increase the susceptibility of encountering *N. fowleri* [5,6,7]. Once encountered, an individual may obtain the CNS infection PAM, with a mortality rate exceeding 90%; this infection is not limited to immuno-compromised patients only, but immuno-competent patients may also come across it [6,8,9,10,11,12,13,14]. Unfortunately, as temperatures are on a rise due to global warming, meaning extremely hot summers are more frequent, an increase in the number of encephalitis cases has been found; in fact, the number of reported cases of PAM has increased globally [2,6,7,15,16]. 

Making matters worse is the lack of effective medications and awareness towards this free-living amoeba [15]. The drug of choice toward the treatment of PAM is the anti-fungal amphotericin B [1,10,15,17,18,19]. Amphotericin B is believed to alter the membrane permeability by binding to ergosterol within the cell membrane and to create pores, leading to the cell death [1,10]. However, amphotericin B has been associated with nephrotoxicity, as a high concentration of it is necessary in order to pass through the blood–brain barrier; hence, the development of new and safe drugs is necessary [10,12,15,19]. In this study, a series of imidazothiazole derivatives previously reported as antiproliferative agents were tested against *N. fowleri* for their anti-amoebic, cytotoxic, and cytopathogenic properties.

We previously reported a series of imidazo[2,1-*b*]thiazole derivatives as anti-proliferative candidates against melanoma, as well as other types of cancer [20,21,22,23,24]. They act via the inhibition of RAF kinases, including V600E-B-RAF and C-RAF. They are able to penetrate the cell membrane and inhibit the kinase inside melanoma cells. In addition, this series of compounds showed promising anti-amoebic activity against *Acanthamoeba castellanii* [25]. This encouraged us to extend our anti-amoebic investigations of the compounds with the hope of finding promising leads. Therefore, we decided to test their anti-amoebic activity against *N. fowleri* based on their cell-membrane-penetrant properties.

## 2. Results

### 2.1. Majority of Compounds Exhibited Significant Amoebicidal Properties against Naegleria fowleri

**Figure 1:**
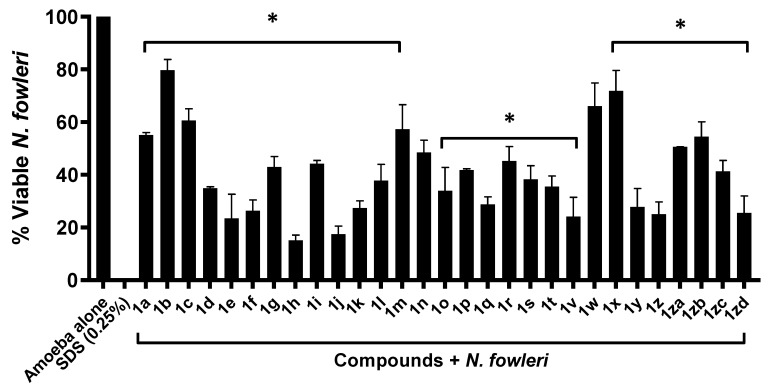
The majority of drugs exhibited significant amoebicidal properties against *Naegleria fowleri.* Significant anti-amoebic effects were found for the majority of compounds against *N. fowleri* after a 24 h incubation period. The data are illustrative of independent experiments performed and are presented with a mean ± standard error. Furthermore, the *p*-values were determined through the conduction of a two-sample *t*-test with two-tailed distributions (* is ≤ 0.05).

The antiamoebic properties of the compounds against *N. fowleri* were determined through amoebicidal assays. After the incubation of *N. fowleri* with 50 µM of the compounds, the amoebicidal activity levels of the compounds were recorded, and those exhibiting significant activity were determined. According to the results obtained, most compounds showed statistically significant amoebicidal properties (*t*-test, two-tailed distribution, *p* ≤ 0.05); of the various compounds tested, only the compounds **1n** and **1w** exhibited no significant effect (Figure 1)*,* whereas **1a, 1d, 1e, 1i, 1f, 1g, 1h, 1j, 1k, 1l, 1o, 1p, 1q, 1r, 1s, 1t, 1v, 1zc, 1zd, 1m, 1c, 1b, 1zb, 1z, 1y, 1za,** and **1x** decreased the amoeba’s viability significantly from 100% to 55.12%, 34.84%, 23.46%, 44.21, 26.41%, 42.94%, 15.14%, 17.45%, 27.39%, 37.75%, 33.97%, 41.77%, 28.78%, 45.23%, 38.29%, 35.54%, 24.13%, 41.31%, 25.54%, 57.28%, 60.64%,79.72%, 54.45%, 71.91%, 25.08%, 50.59%, and 27.82%, respectively (Figure 1).

Additionally, the minimum inhibitory concentrations required to inhibit 50% of *N. fowleri* growth (MIC_50_) of compounds for **1e**, **1f**, and **1h** were determined through the conduction of amoebicidal assays at concentrations of 25 µM, 50 µM, and 75 µM (Table 1). According to the results obtained, at concentrations of 50.96 µM, 60.87 µM, and 48.45 µM, compounds **1e**, **1h**, and **1f** are capable of inhibiting 50% of *N. fowleri* growth (Table 1).

### 2.2. Compounds Exhibited Limited Cytotoxicity against Human Cell Lines

**Figure 2:**
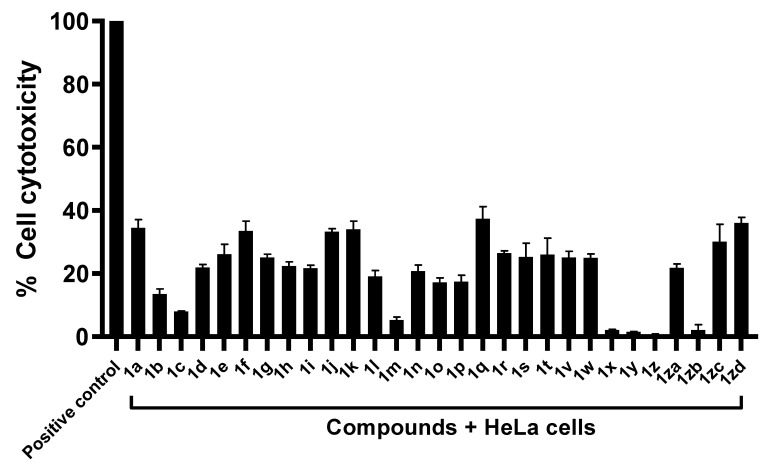
The majority of compounds exhibited minimal cytotoxicity against human cell lines. Confluent monolayers of HeLa cells were treated with 50 µM of the compounds. The compounds **1o, 1l, 1p, 1m, 1c, 1b, 1zb, 1z, 1y**, and **1x** were non-toxic, whereas the compounds **1r, 1s, 1t, 1zc, 1q, 1n, 1a, 1h, 1i, 1f, 1g, 1e, 1j, 1d, 1k, 1v, 1za, 1w,** and **1zd** exhibited limited cytotoxicity against human cells.

The toxicity levels of the compounds against human cells were determined through the conduction of LDH assays. As per ISO 10993-5, if 80% of the cells are viable, the compounds are considered to be non-toxic, whereas if the cell viability is between 40% and 60%, the compounds are considered to be weakly toxic [26,27]. Hence, the compounds **1r, 1s, 1t, 1zc, 1q, 1n, 1a, 1h, 1i, 1f, 1g, 1e, 1j, 1d, 1k, 1v, 1za, 1w,** and **1zd** exhibited limited cytotoxicity against human cells, as they were found to exhibit 26.5%, 25.3%, 26.1%, 30.1%, 37.4%, 20.8%, 34.4%, 22.4%, 21.7%, 33.6%, 25.1%, 26.1%, 33.3%, 22%, 34.1%, 25.1%, 21.8%, 24.9%, and 36.1% cell viability, respectively (Figure 2). The compounds **1o, 1l, 1p, 1m, 1c, 1b, 1zb, 1z, 1y**, and **1x** were found to be non-toxic, possessing minimal toxicity at 15.4%, 17.2%, 19.1%, 17.5%, 5.3%, 7.9%, 13.5%, 2.2%, 0.8%, 1.5%, and 2.2%, respectively (Figure 2).

### 2.3. Majority of Compounds Resulted in Decreased in Amoeba-Mediated Cytotoxic Activity against Human Cells

**Figure 3:**
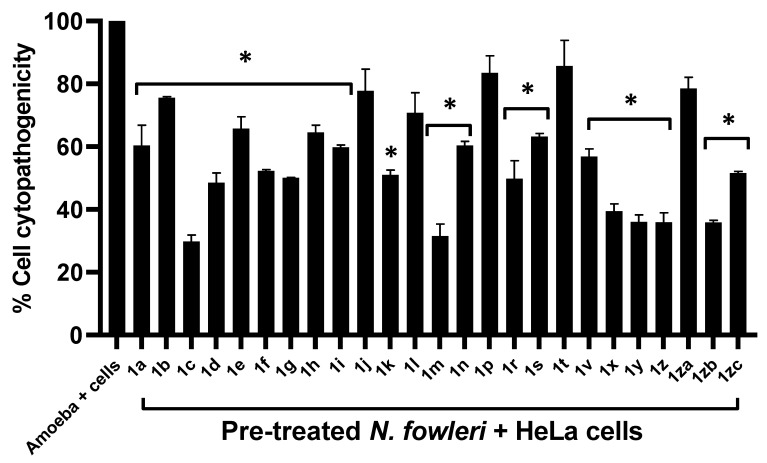
The majority of compounds resulted in decrease in amoeba-mediated cytotoxic activity against human cells. Here, 50 µM of the compounds reduced the *Naegleria fowleri*-mediated cytotoxicity against human cells; 2 × 10^5^ *N. fowleri* cells were incubated with 50 µM of the compound for 2 h. Following the incubation period, the pre-treated were amoebae placed on HeLa cell monolayers and incubated overnight. It was found that some of the compounds were capable of inhibiting the amoeba-meditated host cytotoxicity. * Significant reduction in amoeba-mediated cytotoxicity compared to the untreated amoeba + cells.

The amoeba-mediated host cell death was determined through cytopathogenicity assays. Upon the pre-treatment of *N. fowleri* with compounds **1r, 1s, 1t, 1zc, 1n, 1a, 1h, 1i, 1f, 1g, 1e, 1j, 1d, 1k, 1v, 1za, 1l, 1p, 1m, 1c, 1b, 1zb, 1z, 1y**, and **1x**, reductions in amoeba-mediated cell toxicity were found. The compounds reduced the toxicity of *N. fowleri* against human cells from 100% to 49.8%, 63.2%, 85.7%, 51.6%, 60.4%, 60.4%, 64.5%, 59.8%, 52.3%, 50.1%, 65.8%, 77.8%, 48.5%, 51%, 56.9%, 78.5%, 70.8%, 83.5%, 31.6%, 29.8%, 75.7%, 35.9%, 35.9%, 36.1%, and 39.4%, respectively (Figure 3).

## 3. Discussion

*N. fowleri* is a free-living, unicellular, eukaryotic protist found across the environment [14,28,29,30,31]. This amoeba is responsible for the fatal CNS infection PAM, which has a mortality rate exceeding 90% [29,31,32,33]. Making matters worse are the increasing temperatures due to global warming, as this amoeba prefers warmer temperatures [7,15]. Additionally, individuals pursuing water-related activities such as diving, swimming, nasal cleansing, and ritual ablutions are more susceptible to encountering this amoeba and being infected [6,7]. However, no effective treatment is available, as the drug of choice for the treatment of PAM is amphotericin B, which needs to be administered in high doses to be able to pass the blood–brain barrier, meaning side effects such as nephrotoxicity may result [15,17,18,19].

As the search for safer and improved treatments against *N. fowleri* infection continue, various studies have been conducted. For example, recent studies involved the conjugation of silver and gold nanoparticles with various drugs to enhance their efficacy, with the aim of enhancing the efficacy of the available treatments [11,15,33]. Another study observed the effect of cholesterol-lowering statins against *N. fowleri* [34]. Pavastatin was found to show potent amoebicidal activity against *N. fowleri* strains [34].

In this study, the tested synthetic compounds were hypothesized to show significant anti-amoebic properties. The anti-amoebic properties of the compounds were determined through the conduction of amoebicidal and cytopathogenicity assays; furthermore, the safety of the compounds against human cells was determined through the conduction of lactate dehydrogenase assays through which the cell cytotoxicity levels of the compounds were determined. Of interest are the compounds **1k**, **1f**, and **1v**, as they are capable of reducing amoeba-mediated host cell death to 51%, 52.3%, and 56.9%, respectfully; additionally, these compounds exhibit amoebicidal properties as well, whereby the *N. fowleri* viability was found to be reduced to 27.39%, 26.41%, and 24.13%, respectively. Moreover, the compounds **1c** and **1d** were found to induce the greatest reductions in amoeba-mediated host cell death, reducing the cell death to 29.8% and 48.5%, respectfully. Additionally, the minimum inhibitory concentrations required to inhibit 50% of *N. fowleri* growth for the compounds **1e**, **1h**, and **1f** were determined to be 50.96 µM, 60.87 µM, and 48.45 µM, respectfully. Furthermore, the majority of the compounds were found to possess weak cytotoxicity, except for compounds **1o, 1l, 1p, 1m, 1c, 1b, 1zb, 1z, 1y**, and **1x**, as they were found to possess minimal toxicity. However, studies are needed to determine the effects of selective compounds on both primary as well as immortalized cells. In this study, HeLa cells were employed, since they are an affordable, simplistic model, are widely utilized in biomedical research, and are easy to culture. Future studies are needed to determine the efficacy of the aforementioned compounds against amoeba-mediated host cell damage against the primary brain microvascular endothelial cells that constitute the blood–brain barrier and inhibit drug transport into the brain, as well as in vivo studies to determine whether these compounds can retain their efficacy in animal models of primary amoebic meningoencephalitis due to *N. fowleri*.

Since the anti-amoebic properties of the compounds against *N. fowleri* trophozoites were determined, future studies studying the anti-amoebic properties of the compounds against *N. fowleri* cysts should be conducted. Encystation and excystation assays must be conducted to understand the effects of the compounds in cyst inducement and cyst prevention. Moreover, the mechanism of action of the compounds is unknown. The compounds may be impacting the amoeba membrane or inducing apoptosis; this can be determined through conducting an assay and observing the anti-amoebic effect using an electron microscope.

After conducting a series of in vitro studies, the most promising compounds against *N. fowleri* should be tested in vivo to proceed further with the lead development process. Several studies have reported animal models of the parasite [35,36], which can be considered by researchers working on anti-Naegleria drug discovery.

## 4. Materials and Methods

### 4.1. The Tested Compounds

The series of compounds illustrated in Table 2 were tested in this study.

### 4.2. Henrietta Lacks (HeLa) Cervical Cancer Cells

ATCC CCL-2 Singapore strains of HeLa cells obtained from the American Type Culture Collection (ATCC) were used to maintain *N. fowleri* and to conduct cytotoxicity and cytopathogenicity assays [12].

Within the complete medium, the cells were grown and cultured. The complete media is made from Roswell Park Memorial Institute medium (RPMI), 10% fetal bovine serum (FBS), 1% minimum essential medium amino acids, 1% L-glutamine, and 1% penicillin–streptomycin (Mungroo, et al., 2020). The flasks of the cells were then placed in a 95% humidifying incubator with 5% CO_2_ at 37 °C [12].

### 4.3. Naegleria Fowleri Culture

HB1 ATCC 30174 strains of *N. fowleri* were placed on HeLa cell (ATCC CCL-2) monolayers [12]. The HeLa cell monolayers served as a food source for the amoeba [12]. In a 95% humidifying incubator with 5% CO_2_ and 95% at 37 °C, the *N. fowleri* cultures were maintained. After 48 h, the amoeba was found to have fully consumed the cell monolayers, resulting in an increase in their number, with approximately 5 × 10^5^ being present, from which 95% were in the trophozoite form [11]. Next, the flasks were placed on ice for 15 min to detach the amoebae from the flasks. The media containing amoebae were collected in a 50 mL tube and centrifuged at 2500× *g* for 10 min at 4 °C. Next, the supernatant was discarded and the pellet was resuspended in 1 mL of RPMI. Finally, the amoebae were enumerated and used for subsequent assays.

### 4.4. Amoebicidal Assay

Here, 2 × 10^5^ amoebae were treated with different compounds at 50 µM concentrations and placed in a 96-well plate to determine the amoebicidal properties of the compounds [12]. After treatment, the 96-well plate was placed in an incubator containing 95% humidity and 5% CO_2_ at 37 °C for 24 h.

The following controls were utilized in the assays: amoebae alone with RPMI was the negative control, while amoebae treated with 0.25% SDS was the positive control. Live and dead amoebae were distinguished by the addition of 0.1% methylene blue [12]. The viable amoebae were then counted using a hemocytometer [12].

Furthermore, a student’s *t*-test with a two-tailed distribution was conducted to determine the statistically significant compounds [12]. Furthermore, the MIC_50_ values of the compounds were determined using concentrations of 25 µM, 50 µM, and 75 µM [27].

### 4.5. Cytotoxicity Assay

The HeLa cells were cultured in 96-well plates and treated with different compounds to determine the toxicity of the compounds against the cells. The treated cells were then placed in an incubator with 95% humidity and 5% CO_2_ at 37 °C for 24 h [12]. Following the overnight incubation period, the cell-free supernatant was gathered and the cytotoxicity of the compounds was determined. This was done through the use of a cytotoxicity detection kit, which measures the quantity of lactate dehydrogenase (LDH) release [37].

The inclusion of positive and negative controls insured accurate results. Serving as the negative control were untreated HeLa cells alone; serving as the positive control were HeLa cells treated with 1% Triton X-100.

Furthermore, by carrying out all of the necessary calculations, the cytotoxic properties of the compounds were determined: (absorbance of media from cells treated with the drugs—absorbance of media from cells of negative control)/(absorbance of media from cells of positive control—absorbance of media from cells of negative control) × 100 [12].

### 4.6. Cytopathogenicity Assay

Here, 2 × 10^5^ *N. fowleri* cells were treated with 50 µM of the compounds to determine the amoeba-mediated host cell death. After treatment, the amoebae were incubated for 2 h at 37 °C with 5% CO_2_ and 95% humidity. Next, the treated *N. fowleri* cells were placed in 96-well plates containing HeLa cell monolayers overnight [12]. The following day, the supernatant was collected and the cytotoxicity was determined following the method and calculations described earlier [12].

Positive and negative controls were added to the assay to ensure accurate results were obtained. Untreated *N. fowleri* on HeLa cells served as the negative control, while cells treated with Triton X-100 served as the positive control [12].

### 4.7. Statistical Analysis

All results presented are descriptive of the mean ± standard error from various independent experiments. Additionally, the statistical significance was determined via a two-tailed distribution *t*-test [12]. Additionally, to further examine and elaborate the results the *p* values were determined.

## Figures and Tables

**Table 1:** The MIC_50_ values of the compounds against *N. fowleri* were determined for the various compounds against *N. fowleri*. Concentrations of 25 µM, 50 µM, and 75 µM were tested.

*Naegleria fowleri*				
	25 µM	50 µM	75 µM	MIC_50_
Amoeba Alone	100			
**1e**	93 ± 9.7	52.16 ± 1.7	19 ± 1.7	50.96
**1f**	99 ± 7.3	44 ± 3.9	17 ± 4.4	48.45
**1h**	96 ± 8.5	73 ± 2.4	26 ± 0.8	60.87

**Table 2:** Structures of the tested target compounds **1a**–**zd**.

Compound No.	Structure
**1a**	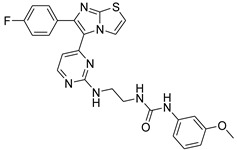
**1b**	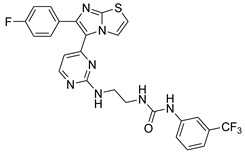
**1c**	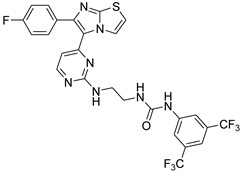
**1d**	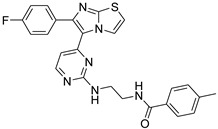
**1e**	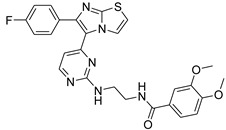
**1f**	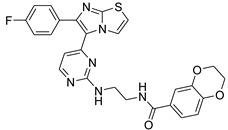
**1g**	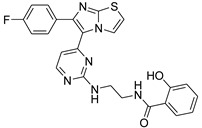
**1h**	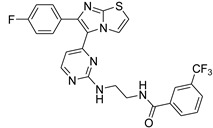
**1i**	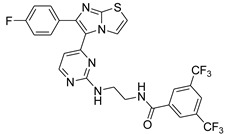
**1j**	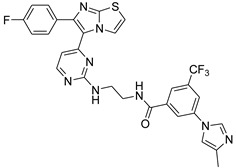
**1k**	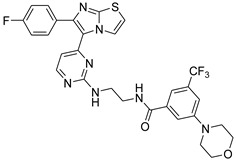
**1l**	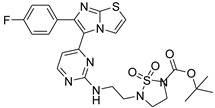
**1m**	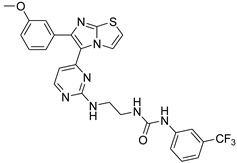
**1n**	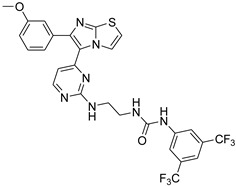
**1o**	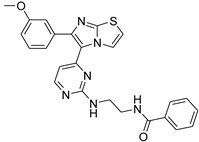
**1p**	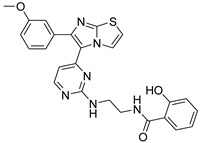
**1q**	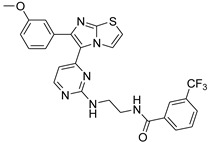
**1r**	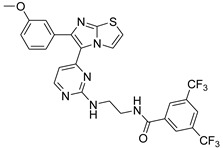
**1s**	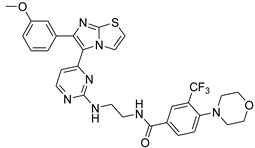
**1t**	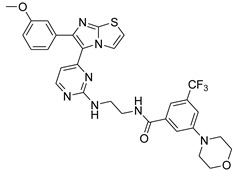
**1u**	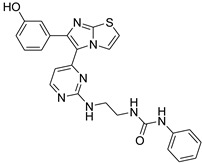
**1v**	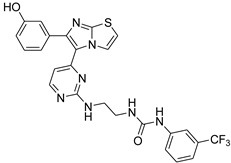
**1w**	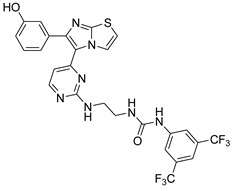
**1x**	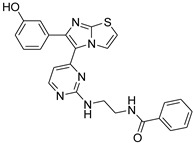
**1y**	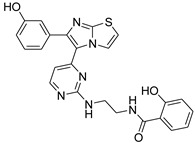
**1z**	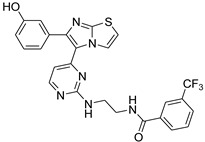
**1za**	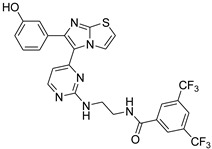
**1zb**	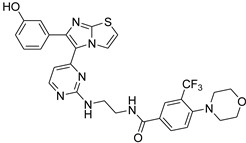
**1zc**	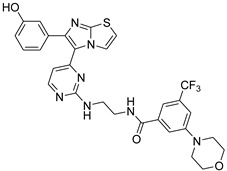
**1zd**	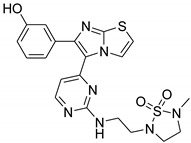

## Data Availability

The datasets generated or analyzed during the current study are available from the corresponding author upon reasonable request.
